# Aging alters circadian regulation of redox in *Drosophila*

**DOI:** 10.3389/fgene.2015.00083

**Published:** 2015-03-09

**Authors:** Vladimir I. Klichko, Eileen S. Chow, Joanna Kotwica-Rolinska, William C. Orr, Jadwiga M. Giebultowicz, Svetlana N. Radyuk

**Affiliations:** ^1^Department of Biological Sciences, Southern Methodist University, DallasTX, USA; ^2^Department of Integrative Biology, Oregon State University, CorvallisOR, USA

**Keywords:** aging, circadian clocks, redox, glutathione, *Drosophila*

## Abstract

Circadian coordination of metabolism, physiology, and neural functions contributes to healthy aging and disease prevention. Clock genes govern the daily rhythmic expression of target genes whose activities underlie such broad physiological parameters as maintenance of redox homeostasis. Previously, we reported that glutathione (GSH) biosynthesis is controlled by the circadian system via effects of the clock genes on expression of the catalytic (*Gclc*) and modulatory (*Gclm*) subunits comprising the glutamate cysteine ligase (GCL) holoenzyme. The objective of this study was to determine whether and how aging, which leads to weakened circadian oscillations, affects the daily profiles of redox-active biomolecules. We found that fly aging is associated with altered profiles of *Gclc* and *Gclm* expression at both the mRNA and protein levels. Analysis of free aminothiols and GCL activity revealed that aging abolishes daily oscillations in GSH levels and alters the activity of glutathione biosynthetic pathways. Unlike GSH, its precursors and products of catabolism, methionine, cysteine and cysteinyl-glycine, were not rhythmic in young or old flies, while rhythms of the glutathione oxidation product, GSSG, were detectable. We conclude that the temporal regulation of GSH biosynthesis is altered in the aging organism and that age-related loss of circadian modulation of pathways involved in glutathione production is likely to impair temporal redox homeostasis.

## Introduction

A growing body of evidence suggests that circadian coordination of metabolism, physiology, and neural functions contributes to healthy aging and disease prevention (Reddy and O’Neill, 2010). Indeed, genetic or environmental disruption of circadian rhythms has been shown to lead to premature aging and age-related pathologies (Kondratova and Kondratov, 2012). From a molecular perspective, the clock genes encode a set of transcriptional regulators whose daily fluctuations dictate the rhythmic expression of their target clock-controlled genes (CCGs). Several genome-wide studies performed around the clock suggest that a substantial fraction of genes are expressed rhythmically in different tissues of flies and mice (Hughes et al., 2012; Koike et al., 2012; Rodriguez et al., 2013). CCGs often constitute a rate-limiting step in pathways involved in metabolism, energy balance, DNA-damage repair, and xenobiotic detoxification in both mammals (Claudel et al., 2007; Green et al., 2008; Kang et al., 2009) and *Drosophila* (Hooven et al., 2009; Beaver et al., 2010; Xu et al., 2011). Moreover, clock gene mutations have been shown to have negative consequences in various biological processes and to shorten life span (Kolker et al., 2004; Khapre et al., 2010; Robertson and Keene, 2013). For example, mice lacking the clock protein BMAL1 (homolog of fly CYC protein) display several symptoms of aging (Kondratov et al., 2006) and show increased neurodegeneration (Musiek et al., 2013). In flies, a null mutation in the clock gene *period* (*per*) leads to shortened life span, accelerated functional decline and increased neuronal degeneration during aging, accompanied by faster and more pronounced accumulation of oxidative damage (Krishnan et al., 2009, 2012). While the connection between the circadian system and aging is relatively well-established, efforts to discern the molecular mechanisms underlying these connections have only recently been initiated.

One of the emerging roles of circadian clocks concerns the regulation of antioxidant defenses and cellular redox (Patel et al., 2014). Studies in *Drosophila* have shown that the levels of reactive oxygen species (ROS) and oxidatively damaged (carbonylated) proteins fluctuate in a daily rhythm in heads of wild type flies and that susceptibility to oxidative challenge is gated by the circadian clock (Krishnan et al., 2008). Recently, a clock-gated response to oxidative injury and regulation of the antioxidant genes was also reported in mice (Pekovic-Vaughan et al., 2014). Circadian regulation was also implicated in oxidative metabolism through rhythmic control of NAD^+^ biosynthesis (Peek et al., 2013). Additionally, daily transcriptional rhythms in genes regulating redox and response to oxidative stress have been demonstrated in brain, liver, lungs, and other murine tissues (Wang et al., 2012; Patel et al., 2014). In particular, daily changes in glutathione (GSH) levels, a central player in the antioxidant defense network and redox-sensitive signaling, along with daily changes in the levels of GSH-biosynthetic gene products, were observed in different mammalian organs (Hardeland et al., 2003; Pekovic-Vaughan et al., 2014). These changes were ascribed to clock-gene regulation of GSH synthesis and homeostasis, mediated via the NRF2 signaling pathway (NRF2 signaling; Pekovic-Vaughan et al., 2014) or via the microRNA-controlled rhythms in GSH levels (Kinoshita et al., 2014). We recently reported that the diurnal fluctuations of GSH levels in *Drosophila* were dependent on the rhythmic expression of genes encoding the catalytic (*Gclc*) and modulatory (*Gclm*) subunits of glutamate cysteine ligase (GCL), the rate-limiting enzyme in GSH biosynthesis (Beaver et al., 2012). Furthermore, we reported that the expression of *GstD1*, which utilizes GSH in cellular detoxification, is also controlled by the clock, based on the observation that these rhythms were abrogated in arrhythmic clock gene mutants (Beaver et al., 2012).

It is notable that multiple hypotheses of aging postulate an important role for redox state dynamics, and this involvement is buttressed by several lines of evidence. Thus, over-expression of genes, such as GCL, Glucose 6 Phosphate Dehydrogenase, and Peroxiredoxin 5, foster a pro-reducing cellular environment, enhance GSH production and confer strong positive effects on longevity (Orr et al., 2005; Legan et al., 2008; Radyuk et al., 2009). GSH-dependent detoxification responses mediated by glutathione *S*-transferase (GST) activity have also been shown to promote beneficial longevity effects (Sykiotis and Bohmann, 2008). Despite growing evidence that circadian and antioxidant/redox systems both modulate longevity, the possible connections between these systems remain to be fully understood. In order to gain insights into potential links between the clock, redox and aging, we investigated the fate of redox-related rhythms in aging *Drosophila*.

We determined recently that functional clocks are necessary for rhythmic transcription of *Gclc* and *Gclm* and for rhythmicity in GSH levels in young male flies, as the rhythms of these molecules were abolished in the clock mutants *cyc^*01*^* and *per^*01*^* (Beaver et al., 2012). It was also established that clock gene oscillations become significantly reduced in heads of old males (Luo et al., 2012; Rakshit et al., 2012); however, profiles of CCGs have not been studied during aging. To address this question, we investigated how age-related weakening of the clock gene oscillations affects daily profiles of factors involved in glutathione synthesis and metabolism. We report that the rhythms in cellular redox observed in young flies are significantly altered in the heads of old flies, leading to compromised GSH homeostasis.

## Materials and Methods

### Fly Rearing and Strains

Flies were raised on standard cornmeal-yeast-molasses diet at 25^∘^C under a 12 h light/12 h dark (LD) regimen (where Zeitgeber time (ZT) 0 is time of lights on and ZT12 is time of lights off). Light intensity was kept at ∼1500 lux. Mated males were separated 1–2 days after emergence. Aging males were kept in inverted 8 oz round bottom polypropylene bottles (Genesee Scientific, San Diego, CA, USA) on 35 mm petri dishes (BD Falcon, San Jose, CA, USA) containing 15 ml of diet. Fresh diet was provided three times a week. All experiments were completed using the *w^*1118*^* strain.

### Quantitative Real-Time PCR

Fly heads were separated using metal sieves frozen with liquid nitrogen. RNA was extracted from 50 heads, which were homogenized using a Kontes handheld motor in Trizol (Life Technologies, Grand Island, NY, USA) followed by ethanol precipitation. Samples were treated with DNAse (Takara, Mountain View, CA, USA). DNAse was deactivated by phenol/chloroform extraction, and samples were purified with sodium acetate. Synthesis of cDNA was achieved with iScript cDNA synthesis kit (Bio-Rad, Hercules, CA, USA) according to the manufacturer’s protocol. Quantitative real-time PCR was performed with Power SYBR Green Master Mix (Life Technologies) on an Applied Biosystems Step-One Plus real-time machine. Primers were obtained from IDT Technology (Coralville, IA, USA). All primers used in this study had efficiencies >96%, and their sequences have been published (Beaver et al., 2012). Data were normalized to *rp49* (Ling and Salvaterra, 2011) as indicated in the results and analyzed using the standard 2^-ΔΔCT^ method. Statistics were calculated using GraphPad Prism 6 (San Diego, CA, USA).

### Immunoblot Analysis

Samples were collected at 4 h intervals from at least 10 heads obtained from *w^*1118*^* flies separated using sieves frozen with liquid nitrogen, and processed as described Beaver et al. (2012). Briefly, samples in the amount of ∼5 μg of total protein were resolved by PAGE and the immunoblots were developed with antibodies generated against recombinant GCLc and GCLm proteins (Orr et al., 2005) and anti-actin antibodies (MP Biomedicals, Santa Ana, CA, USA) to control for loading. The intensity of signals was analyzed by densitometric scanning, using the digital imaging analysis system with AlphaEase Stand Alone Software (Alpha Innotech Corp., San Leandro, CA, USA).

### GCL Enzyme Activity and Free Aminothiols Levels

Glutamate cysteine ligase enzyme activity was measured essentially as described Toroser and Sohal (2005) and Beaver et al. (2012). Linearity of time, protein and substrate concentrations were determined in pilot experiments. GCL activity reaction was performed in duplicate immediately after protein preparation, as suggested (Toroser and Sohal, 2005). The reaction was terminated by adding an equal volume of freshly prepared 10% (w/v) meta-phosphoric acid (MPA). The GCL inhibitor l-buthionine-*S*,*R*-sulfoximine was used to determine specificity of the assay. After GCL reaction, samples were immediately analyzed by HPLC or stored at –80^∘^C for no longer than 24 h before analysis.

Free aminothiol content in fly heads was quantified by HPLC as described Melnyk et al. (1999). Briefly, 50 heads were homogenized in 200 μl of freshly prepared ice-cold 5% MPA, incubated for 30 min on ice and centrifuged at 16000 *g* for 20 min at 4^∘^C. Supernatants were filtered through 0.22 μm PTFE membrane syringe filter and immediately analyzed by HPLC or stored at –80^∘^C. The amount of protein in the precipitate was determined using the DC protein assay (Bio-Rad Laboratories, Hercules, CA, USA) according to manufacturer’s recommendations (Bulletin 1770 US/EG Rev A, Bio-Rad).

Preparations from GCL assays and free aminothiol extractions were resolved by HPLC under isocratic elution using a reverse-phase C18 Gemini-NX (3 μm, 4.6 × 150 mm) column (Phenomenex, Torrance, CA, USA) with the flow rate of 0.75 ml/min. The mobile phase contains 2% (v/v) acetonitrile, 25 mM monobasic sodium phosphate, 1.5 mM 1-octane sulfonic acid as ion-pairing agent, pH 2.7, adjusted with ortho-phosphoric acid. Aminothiols were detected using the 5600 CoulArray electrochemical detector equipped with four-channel analytical cell (ESA Inc., Chelmsford, MA, USA). Increasing potentials of +100, +200, +750, +850 mV in channels 1–4, respectively, were used for measuring γ-Glu-Cys in GCL activity assay samples. γ-Glu-Cys was detected in channel 3 at +750 mV. Potentials of +550, +600, +750, and +875 mV were used for detection of aminothiols in extracts. Each sample was injected twice. Calibration standards were prepared in 5% (w/v) MPA and injected at regular intervals.

## Results

### Age-Related Changes in GCLc and GCLm Profiles

**FIGURE 1:**
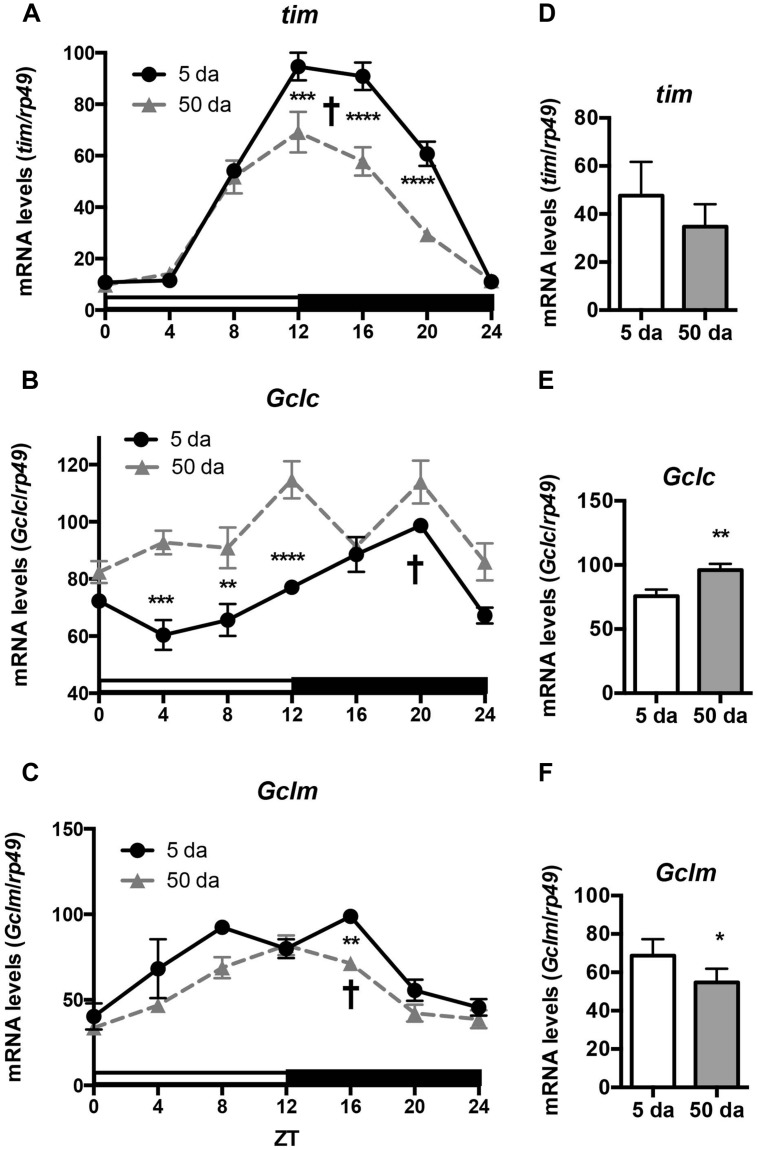
Rhythms in mRNA expression of *Gclc* and *Gclm* show age-related differences. Levels of *tim*, *Gclc*, and *Gclm* mRNA were measured in *w^*1118*^* male heads at ages day 5 and 50 post-eclosion. **(A,D)** Young flies show strong rhythms in transcription of the clock gene *tim*, the rhythm was maintained in old but expression at peak time points was significantly decreased. **(B,E)** Young flies show rhythmic unimodal *Gclc* expression, while in old flies overall mRNA levels are significantly higher at most time points. **(C,F)** Young and old flies show significant rhythms in *Gclm* expression, but overall levels are lower in old. Levels are normalized to the 5 Da peak values set as 100%. Graphics on the right represent average 24-h levels of *tim*, *Gclc,* and *Gclm* mRNA levels in 5 and 50 Da old flies. Significance was analyzed by 2-way ANOVA with Bonferonni post-test; ^∗^ marks a significant difference between day 5 and day 50 (^∗^*p* < 0.05, ^∗∗^*p* < 0.01, ^∗∗∗^*p* < 0.001, ^∗∗∗∗^*p* < 0.0001). † marks a significant difference between minimum and maximum expression in young flies.

**FIGURE 2:**
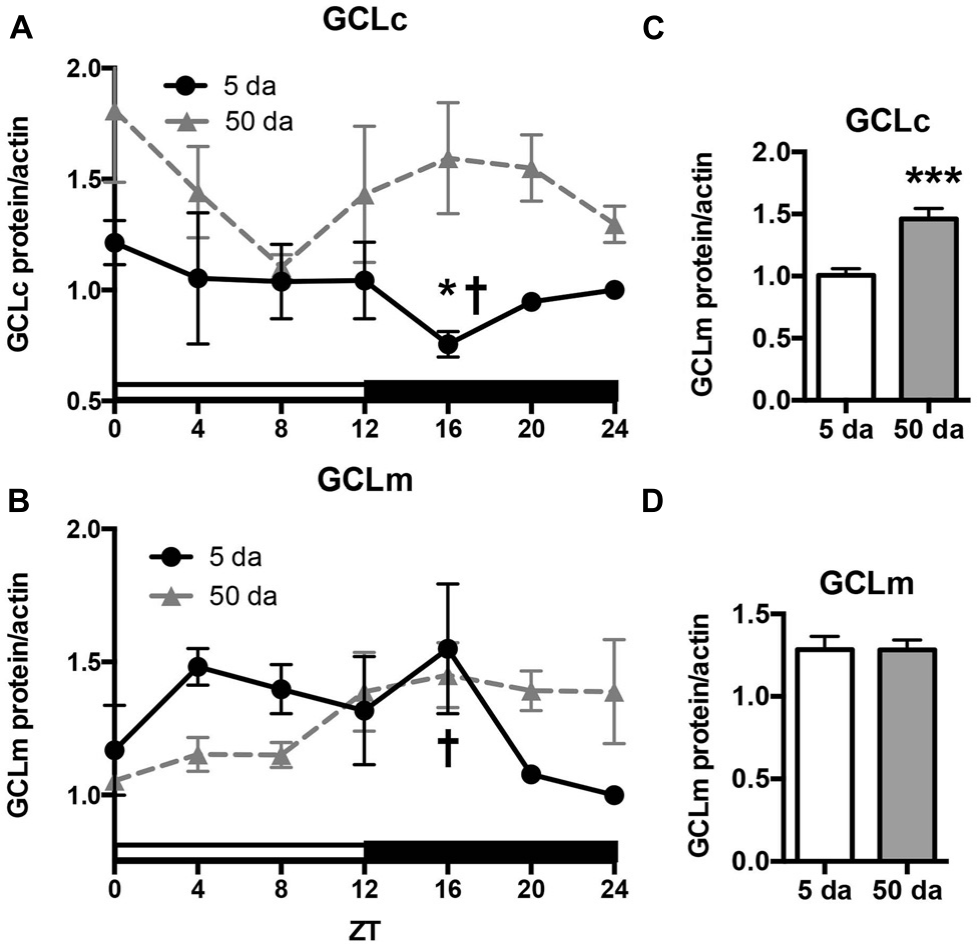
Effects of aging on GCLc **(A,C)** and GCLm **(B,D)** protein levels. Protein lysates were prepared from the heads of young (5 Da) and old (50 Da) *w^*1118*^* males and immunoblotted using anti-GCLc and anti-GCLm antibodies followed by re-probing with anti-actin antibodies to control for loading. Each replicate was normalized to the time point with the lowest expression. Data represent average values ± SEM obtained from three independent bio-replicates. Data were analyzed by a 2-way ANOVA and Bonferroni’s post-test; ^∗^ marks a significant difference between day 5 and day 50 (^∗^*p* < 0.05, ^∗∗∗^*p* < 0.001). Graphics on the right represent average 24-h levels of GCLc **(C)** and GCLm **(D)** proteins in 5 and 50 Da old flies. † marks a significant difference between minimum and maximum expression in young flies.

**FIGURE 3:**
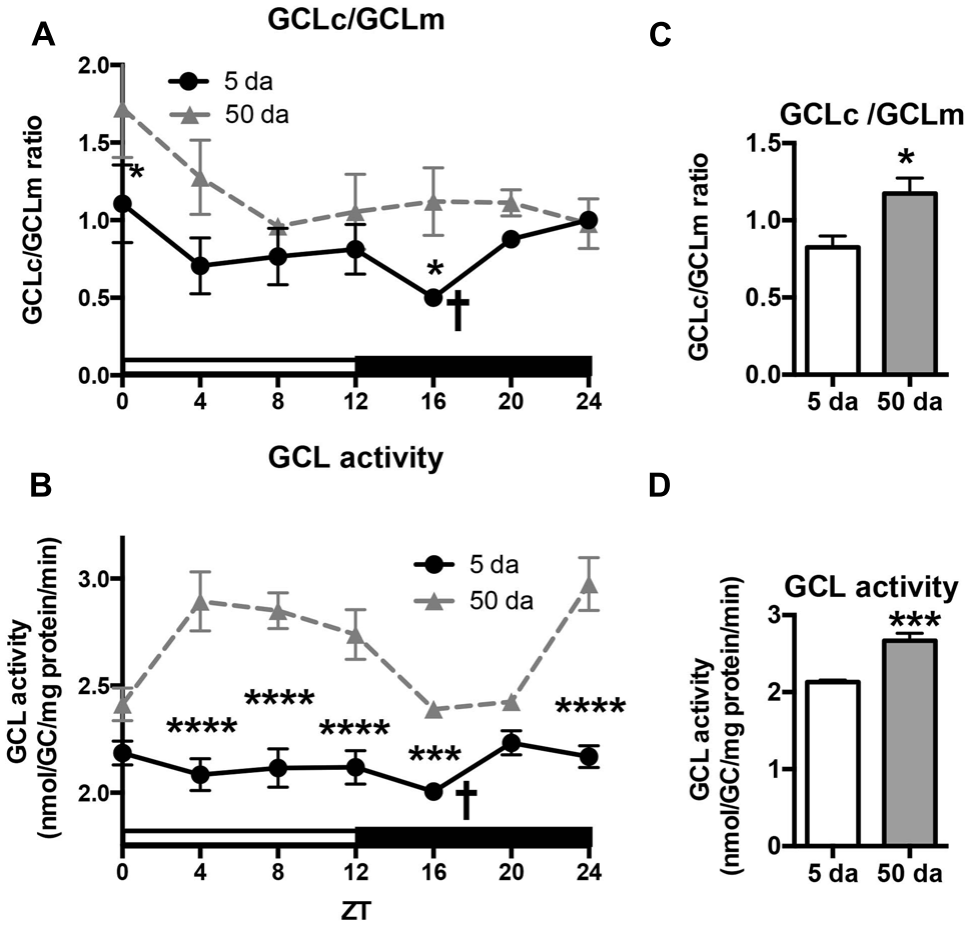
Effects of aging on GCLc to GCLm protein ratio **(A,C)** and activity of GCL holoenzyme **(B,D)** in the heads of young (5 Da) and old (50 Da) flies. Samples were prepared from the heads of *w^*1118*^* males. GCL activity was measured by the formation of the GCL product, *γ*-Glu-Cys (GC). Data represents average values ± SEM obtained from two independent bio-replicates (total *N* = 8). Ratio of GCLc to GCLm proteins was calculated from the results presented in **Figure 2**. Data were analyzed by a 2-way ANOVA and Bonferroni’s post-test; ^∗^ marks a significant difference between day 5 and day 50 (^∗^*p* < 0.05, ^∗∗∗^*p* < 0.001, ^∗∗∗∗^*p* < 0.0001). Graphics on the right represent average 24-h levels of GCLc/GCLm protein ratio **(C)** and GCL activity **(D)** in 5 and 50 Da old flies. † marks a significant difference between minimum and maximum expression in young flies.

To determine how aging affects clock-controlled *Gclc* and* Gclm* transcription, we compared around the clock profiles of these genes in heads of young and old white-eyed flies (*w*, strain *w^*1118*^*). In prior studies involving young Canton S flies we detected strong diurnal patterns in both *Gclc* and *Gclm* mRNA levels and less pronounced, albeit significant rhythmic fluctuations in proteins levels (Beaver et al., 2012). In this study, we confirm that a similar pattern of daily changes in the expression of *timeless* (*tim)*, *Gclc,* and *Gclm* was observed in young *w^*1118*^* flies (**Figure 1**, Beaver et al., 2012). Comparison of young and old ages showed that old flies had a significantly lower peak expression of the clock gene *tim* compared to their young counterparts (**Figure 1A**); this dampening of the amplitude is a typical signature of the aging of the clock mechanism (Rakshit et al., 2012). The young flies had a unimodal rhythm of *Gclc* expression with a distinct peak at ZT20, similar as previously reported in Canton S flies (Beaver et al., 2012). Surprisingly, levels of *Gclc* mRNA were significantly higher in old flies at ZT4, ZT8, and ZT12, thus abolishing the trough that was observed in young flies (**Figure 1B**). The expression of *Gclm* was significantly rhythmic in young flies with elevated expression at ZT 8–16 (**Figure 1C**) similar to that previously reported in Canton S flies (Beaver et al., 2012). In contrast to *Gclc*, expression of *Gclm* did not increase with age, but even slightly decreased reaching significance at ZT16, and the expression remained significantly rhythmic in old flies (**Figure 1C**). Changes in GCLc and GCLm protein expression as well as the GCLc/GCLm ratio in *w* flies paralleled those reported in Canton S flies (**Figures 2A,B** and **3A**, Beaver et al., 2012). Thus, a significant reduction of GCLc was observed at ZT16 in young flies, but old flies showed significantly elevated protein level at this time as well as most other time points (**Figures 2A,C**). In young flies, the levels of GCLm protein were elevated from ZT4 to ZT16, with a significant peak at ZT16, whereas in 50 Da old flies, there was no significant rhythm (**Figures 2B,D**). The relatively weak albeit statistically significant 24-h rhythmic expression patterns of GCLc and GCLm observed in young flies were also reflected in the rhythmic variation in their ratio; likewise absent in old flies (**Figure 3A**).

We next measured the activity of GCL holoenzyme in homogenates of young and old *w* flies collected around the clock. In agreement with GCLc expression patterns (**Figure 2A**) and variations in the GCLc/GCLm ratio (**Figure 3A**), a significant oscillation of GCL enzyme activity was found in young *w* flies (**Figure 3B**) although with a lesser amplitude compared to Canton S flies (Beaver et al., 2012). In old *w* flies, GCL enzyme activity displayed a very different daily pattern than in young flies (**Figure 3B**), and the average enzyme activity was significantly higher (**Figure 3D**). This is consistent with the observation that both GCLc protein levels and the GCLc/GCLm protein ratio exhibited significantly higher levels in the old flies relative to the young ones (**Figures 2A,C** and **3A,C**).

### Circadian Characteristics of Free Aminothiols Determined in Heads of Young and Old Flies

**FIGURE 4:**
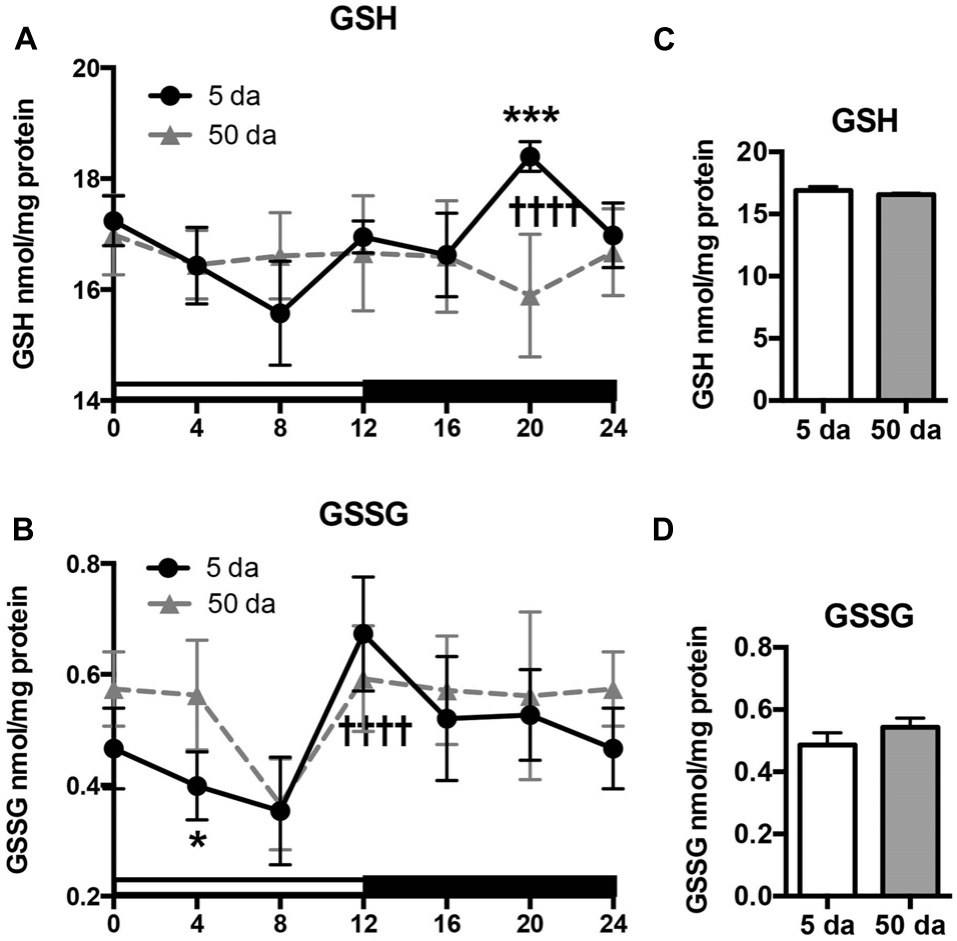
Effects of aging on daily changes in concentrations of GSH **(A,C)** and GSSG **(B,D)** in the fly head homogenates. Samples were made from the heads of young (5 Da) and old (50 Da) *w^*1118*^* males. Results are average values ± SEM obtained from three independent bio-replicates (total *N* = 6). Data were analyzed by a 2-way ANOVA and Bonferroni’s post-test; ^∗^ marks a significant difference between day 5 and day 50 (^∗^*p* < 0.05, ^∗∗∗^*p* < 0.001). Graphics on the right represent average 24-h levels of GSH **(C)** and GSSG **(D)** in 5 and 50 Da old flies. †††† marks a significant difference (*p* < 0.0001) between minimum and maximum expression in young flies.

Previously, we reported that mitochondrial H_2_O_2_ production and GSH concentrations fluctuate in a clock-dependent manner showing opposite profiles; thus at ZT20, the levels of H_2_O_2_ in fly heads reached their lowest point and those of GSH were at their highest, defining a pro-reducing peak, while at ZT8 H_2_O_2_ levels were at their highest and GSH at their lowest (Krishnan et al., 2008; Beaver et al., 2012). Here we show that a significant rhythm in GSH levels is also detected in heads of young *w* flies (**Figure 4A**). In old flies the overall daily levels of GSH were similar to those observed in young flies, although the diurnal oscillations were absent (**Figures 4A,C**). In contrast to GSH, GSSG levels in young flies were relatively low in the morning and continued to decline until ZT8 followed by a significant, approximately twofold increase during the day with a peak at ZT12 (**Figure 4B**). A similar trend was observed in old flies, although the morning GSSG levels did not drop-off as much as they did in young flies, giving rise to a slight albeit non-significant elevation in overall GSSG levels in the heads of aging flies (**Figures 4B,D**, Beaver et al., 2012).

**FIGURE 5:**
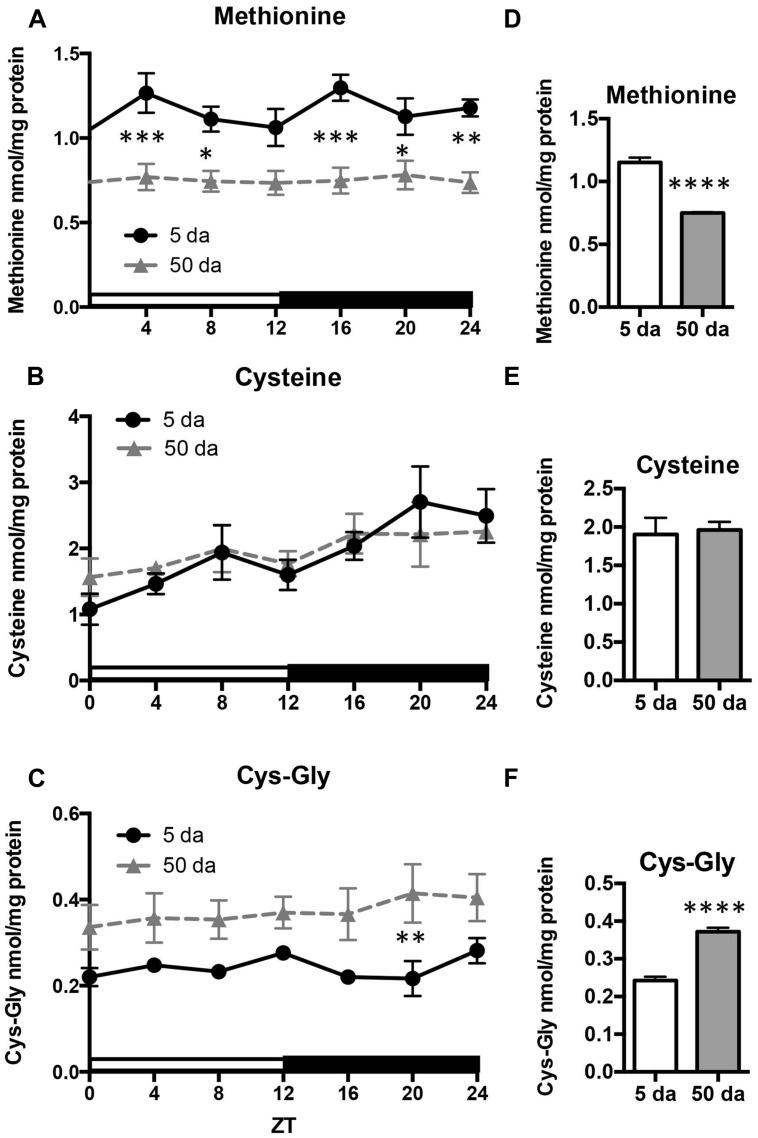
Effects of aging on daily profiles in concentrations of methionine **(A,D)**, cysteine **(B,E)** and Cys-Gly **(C,F)** in the fly head homogenates. Samples were made from the heads of young (5 Da) and old (50 Da) *w^*1118*^* males. Results are average values ± SEM obtained from three independent bio-replicates (total *N* = 6). Data were analyzed by a 2-way ANOVA and Bonferroni’s post-test; ^∗^ marks a significant difference between day 5 and day 50 (^∗^*p* < 0.05, ^∗∗^*p* < 0.01, ^∗∗∗^*p* < 0.001, ^∗∗∗∗^*p* < 0.0001). Graphics on the right represent average 24-h levels of methionine **(D)**, cysteine **(E)**, and Cys-Gly **(F)** in 5 and 50 Da old flies.

Given that the rhythmicity of GSH levels in young flies was absent in old flies, we also investigated the daily profiles of aminothiols, that are involved in glutathione synthesis and metabolism. Changes in methionine, cysteine, and cysteinyl-glycine (Cys-Gly) levels were measured in heads of *w* flies collected around the clock. Unlike GSH, the glutathione precursors, cysteine and methionine, as well as Cys-Gly, which is a break-down product of GSH, did not display rhythmic fluctuations in either young or old flies (**Figure 5**). On the other hand, there were statistically significant age-related differences in these compounds. Levels of free methionine were significantly lower around the clock in old flies (**Figures 5A,D**) while levels of cysteine remained unchanged (**Figures 5B,E**). In contrast to methionine, the levels of Cys-Gly, were significantly higher in the heads of old flies (∼64%, **Figures 5C,F**).

## Discussion

In this study we investigated a role for the circadian system in regulating redox in the context of organismal aging. We have determined that the temporal regulation of the redox-active biomolecules, which is modulated by the circadian clocks in young flies, is significantly altered in old flies with dampened clocks. Our results demonstrate that the circadian clocks regulate redox homeostasis via their effects on glutathione biosynthetic pathways, specifically via transcriptional control of the genes involved in *de novo* GSH synthesis, and that age-related reduction of the circadian clock oscillations in old flies is associated with compromised redox rhythms.

**FIGURE 6:**
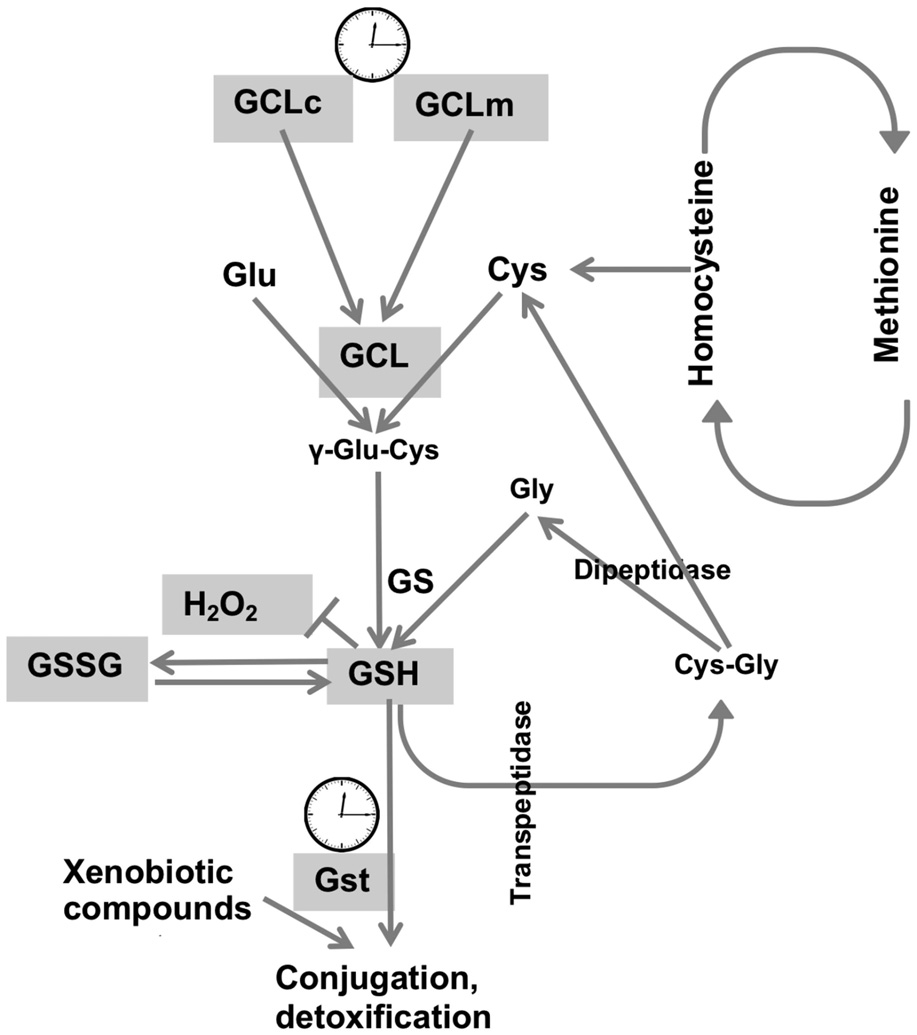
Synthesis and metabolism of glutathione and its regulation by the circadian system. GSH synthesis occurs via two sequential reactions: (i) ligation of l-glutamate and l-cysteine is catalyzed by the activity of GCL, and (ii) the addition of glycine to γ-glutamylcysteine by the activity of glutathione synthetase. GCL is a heterodimer composed of a catalytic subunit (GCLc) and a modulatory subunit (GCLm). Degradation of GSH involves cleavage by *trans*-peptidase, followed by the cleavage of cysteinyl-glycine by a dipeptidase, forming Cys-Gly and cysteine respectively. Cysteine is supplied via *trans*-sulfuration pathway and as a product of GSH degradation. GSH is oxidized by H_2_O_2_ to its disulphide, GSSG. Methionine is the precursor for cysteine synthesis, in which homocysteine is an intermediate. GSH is also used for detoxification in reactions catalyzed by GSTs, which expression is also rhythmic (Beaver et al., 2012). A clock image denotes genes regulated by the circadian clocks. Cycling components of the glutathione synthesis and utilization systems are shown in gray.

Cellular redox homeostasis largely relies on the redox-active compound, glutathione, which is present at concentrations many fold higher compared to concentrations of other redox-active molecules (Rebrin et al., 2004). We had previously shown that the circadian clocks modulate the *de novo* synthesis of GSH via transcriptional control of GCLc and GCLm, the subunits that comprise the GCL holoenzyme (Beaver et al., 2012). In this study we broadened the investigation of the relationship between clock and redox to determine the levels of other redox-active molecules, involved in glutathione synthesis and metabolism. Synthesis of the tri-peptide glutathione, composed of glutamate, cysteine and glycine residues, depends not only on the activity of biosynthetic enzymes, but also on the availability of substrates, where cysteine is a limiting factor. The synthesis of cysteine is mediated by the *trans*-sulfuration pathway, using methionine as the source (Vitvitsky et al., 2003), and this pathway is also active in flies (Kabil et al., 2011). Consequently, we investigated around the clock expression profiles of both cysteine and its precursor methionine. As neither cysteine nor methionine exhibited evidence for diurnal rhythms in either young or old flies, it appears that the contribution of the *trans*-sulfuration pathway to glutathione homeostasis is not regulated by circadian clocks. Consistent with our findings, methionine was found to be arrhythmic in the study of the human metabolome of blood plasma and saliva (Dallmann et al., 2012) although the analyses were performed with healthy but older (57–61 years) males, where age-dependent effects might have influenced the oscillations. In contrast, mouse hepatic metabolome and transcriptome studies revealed rhythmicity in metabolic sub-pathways, where oscillations in glutathione were ascertained by oscillations in its precursors, cysteine and methionine, albeit with a lower amplitude for the latter (Eckel-Mahan et al., 2012). In the same study, hepatic rhythmicity was also observed in the concentrations of Cys-Gly, which peaked at 9 h together with cysteine and methionine. In contrast, analysis of the *Drosophila* heads revealed no cycling in the concentrations of Cys-Gly (**Figure 5C**), consistent with the arrhythmic behavior of cysteine and methionine (**Figures 5A,B**). Given that Cys-Gly also serves as a signature of glutathione degradation, interpretation of these results are somewhat tentative. Nevertheless, our results revealed no rhythmicity in cysteine, methionine and Cys-Gly, and suggest that, at least in flies, the pathways responsible for the supply of sulfur-containing precursors for glutathione synthesis are not regulated by the circadian clocks (**Figure 6**). It should be noted that the mammalian liver is a homogenous tissue with a strong food-entrained clock mechanism, while fly heads are enriched in nervous tissues with clocks entrained by light-dark cycles. Moreover recent analysis of the circadian transcriptome shows that liver possesses the highest number of rhythmic genes, while brain has the lowest (Zhang et al., 2014).

Another important finding of our study is that the diurnal fluctuations in GSH levels were not followed by similar changes in the products of its degradation (Cys-Gly) and oxidation (GSSG). While Cys-Gly was completely arrhythmic (**Figure 5C**), changes in GSSG profile did not mirror those observed for GSH (**Figures 4A,B**). Even though both shared the same slow drop-off from ZT0 to ZT8 as well as the ZT8 trough, their peaks were quite distinct (ZT12 for GSSG and ZT20 for GSH). Also in old flies, a certain degree of rhythmicity is maintained for GSSG in contrast to the absence of any diurnal GSH patterns.

As mentioned above, circadian oscillations of GSH and GSSG in the mouse liver were recently reported and displayed an opposing pattern at ZT9 where GSH exhibited a trough and GSSG exhibited a peak (Eckel-Mahan et al., 2012); the decrease in GSH levels was assumed to come largely at the expense of GSSG formation. Our results suggest that this is not the case in *Drosophila* heads, although the observed differences may be related to the specificity of phase of rhythms in different tissues of different species. This differential oscillation of GSH and GSSG also raises some doubt about the functional relevance of the GSH:GSSG ratio and the redox potential in mediating redox-dependent responses, which was recently critically discussed (Flohe, 2013). Indeed, the ratio of GSSG and GSH may reflect little else than the steady state levels of these molecules, which are maintained by independent enzymatic processes of GSH metabolism and synthesis. In this light, concentrations of GSH account for more than 97% of the total non-protein glutathione pool, while GSSG constitutes only ∼3% (**Figure 4**). Consequently, the observed circadian changes in GSSG levels would constitute a trivial change in GSH levels, suggesting that, rather than mediating redox signaling, GSH is predominantly used by antioxidant and detoxification enzymes, e.g., GSTs and peroxidases, as well as in reactions of protein modification (glutathionylation; Manevich et al., 2004; Tew and Townsend, 2012; Zhang and Forman, 2012; Janssen-Heininger et al., 2013).

Another important finding of this study is that the rhythms in glutathione levels observed in young flies were lost in old flies (**Figure 4**), presumably due to the loss of diurnal fluctuations of GCLc, GCLm as well as GCL activity, in response to the weakening of the circadian clocks (**Figures 1–3**). In contrast, GSSG rhythms were largely preserved in older flies, suggesting that the daily changes in glutathione disulfide levels are supported by enzymatic reactions that are not under clock control.

Despite loss of circadian regulation, average daily levels of GSH remained unchanged during aging (**Figure 4C**), while the levels of GSSG were slightly higher, mainly due to lesser drop in the early morning (**Figures 4B,D**). In *Drosophila*, it has been established that whole body GSH levels were either relatively constant (Rebrin et al., 2004) or slightly decreased during aging while GSSG rose 2–3 fold (Rebrin and Sohal, 2006). Similar age-related changes were documented in different mammalian tissues with the most significant reduction in GSH and accumulation of GSSG in the brain, indicative of a more pro-oxidative cellular environment (Suh et al., 2004b; Zhu et al., 2006; Rebrin and Sohal, 2008). As such changes in GSH and GSSG were frequently associated with increases in enzyme activities related to GSH usage, the relatively steady glutathione concentrations observed in the heads of old flies could point to less efficient GSH utilization.

The rather unexpected finding of our study is that the expression of Gclc at both mRNA and protein levels significantly increased in the heads of old flies, and this increase was associated with about 25% higher average daily GCL activity. Despite this increase, the average daily levels of GSH remained unchanged suggesting a loss in GCL catalytic efficiency or an age-related increase in GSH utilization. One possible scenario is that the efficiency of GSH synthesis can be induced by oxidative stress, in part through the well-documented increase in H_2_O_2_ signaling that accompanies aging (Sohal and Dubey, 1994; Chen et al., 2005; Franklin et al., 2009). For instance, post-translational control of γ-glutamylcysteine (γ-Glu-Cys) synthesis is influenced by oxidative stress, which can dramatically affect formation of GCL holoenzyme and its stabilization (Franklin et al., 2009; Krejsa et al., 2010). Consistent with induction of GCL by stress, we previously reported that *per*-null mutants with disrupted clock displayed arrhythmic as well as elevated GCL activity (Beaver et al., 2012), which was also reflected in arrhythmic and elevated ROS levels relative to the control (Krishnan et al., 2008). It should be noted that previous studies comparing GCL activity and GSH levels in young and old rats showed a decrease of both parameters in liver (Suh et al., 2004b), while in aging brain and heart GSH decreased but GCL activity remained unchanged (Suh et al., 2004a), pointing again to catalytic deficiency of the enzyme.

Other aminothiols that did not show cycling in young flies also remained arrhythmic in old flies, but displayed changes in their steady state levels. Consistent with previous reports, the amounts of Cys-Gly were ∼50% higher in older flies (Rebrin et al., 2004; Rebrin and Sohal, 2006, 2008). Cys-Gly, derived from the breakdown of glutathione, is required for GSH synthesis as a precursor of cysteine, but at the same time it is also a prooxidant generated during the catabolism of glutathione. The requirement of Cys-Gly for GSH synthesis justifies its increase with age, as the tissues require an increased supply of precursors for GSH biosynthesis in older flies. However, we did not observe an increase in cysteine levels during aging. A more plausible explanation is that the increase in Cys-Gly is indicative of an increase in oxidative stress and GSH degradation. In agreement with this view, the average daily levels of methionine were about 35% lower in old flies suggesting the likelihood of an increase in oxidation of methionine to methionine sulfoxide by ROS rather than an increase in methionine consumption for cysteine biosynthesis. Together, these changes indicate a shift in redox homeostasis in the heads of older flies, consistent with the earlier reports in whole flies (Rebrin et al., 2004). Similar alterations in the redox components were also indicative of heightened oxidative stress in pathologies like systemic lupus erythematosus (Wu et al., 2012).

To conclude, this study revealed that the age-related dampening of circadian rhythms in clock genes underlie the age-related loss of rhythmicity of cellular redox.

## Author Contributions

Conceived the study and planned experiments: SR, JG, EC. Performed experiments: VK, EC, JK-R SR, JG. Analyzed data and wrote the paper: EC, VK, SR, WO, JG.

## Conflict of Interest Statement

The authors declare that the research was conducted in the absence of any commercial or financial relationships that could be construed as a potential conflict of interest.
